# Designing molecules: directing stem cell differentiation

**DOI:** 10.3389/fbioe.2024.1396405

**Published:** 2024-05-10

**Authors:** Kalaiselvaan Thanaskody, Fajriyah Nur Natashah, Fazlina Nordin, Wan Safwani Wan Kamarul Zaman, Gee Jun Tye

**Affiliations:** ^1^ Centre for Tissue Engineering and Regenerative Medicine (CTERM), Faculty of Medicine, National University of Malaysia, Kuala Lumpur, Malaysia; ^2^ Department of Pharmaceutical Life Sciences, Faculty of Pharmacy, Universiti Malaya, Kuala Lumpur, Malaysia; ^3^ Centre for Innovation in Medical Engineering (CIME), Department of Biomedical Engineering, Faculty of Engineering, Universiti Malaya, Kuala Lumpur, Malaysia; ^4^ Institute for Research in Molecular Medicine (INFORMM), Universiti Sains Malaysia, Gelugor, Malaysia

**Keywords:** design molecules, aptamers, bacteriophage, biological vectors, stem cell differentiation

## Abstract

Stem cells have been widely applied in regenerative and therapeutic medicine for their unique regenerative properties. Although much research has shown their potential, it remains tricky in directing stem cell differentiation. The advancement of genetic and therapeutic technologies, however, has facilitated this issue through development of design molecules. These molecules are designed to overcome the drawbacks previously faced, such as unexpected differentiation outcomes and insufficient migration of endogenous or exogenous MSCs. Here, we introduced aptamer, bacteriophage, and biological vectors as design molecules and described their characteristics. The methods of designing/developing discussed include various Systematic Evolution of Ligands by Exponential Enrichment (SELEX) procedures, *in silico* approaches, and non-SELEX methods for aptamers, and genetic engineering methods such as homologous recombination, Bacteriophage Recombineering of Electroporated DNA (BRED), Bacteriophage Recombineering with Infectious Particles (BRIP), and genome rebooting for bacteriophage. For biological vectors, methods such as alternate splicing, multiple promoters, internal ribosomal entry site, CRISPR-Cas9 system and Cre recombinase mediated recombination were used to design viral vectors, while non-viral vectors like exosomes are generated through parental cell-based direct engineering. Besides that, we also discussed the pros and cons, and applications of each design molecule in directing stem cell differentiation to illustrate their great potential in stem cells research. Finally, we highlighted some safety and efficacy concerns to be considered for future studies.

## 1 Introduction

Stem cells are cells that have the ability to self-renew and differentiate into other types of cells. They have played many roles in development and maintaining the health of an organism. They consist of several levels of differentiation potential, namely, totipotent, pluripotent, multipotent, and unipotent. Besides differentiation potential, the fate of these stem cells is also influenced by the interplay of various external and internal factors, including their microenvironments, or niches which provides specific cues to the cells (Zakrzewski et al., 2019). These regenerative features of stem cells have attracted researchers to explore and utilize them in many ways, especially in treating incurable and degenerative diseases.

Although this field of research has been studied for a long time, there are still many challenges to overcome. Some examples of current limitations in stem cell differentiation include difficulties in controlling differentiation, where attenuation of *SP7* gene responsible for undesired hypertrophic differentiation had compromised bone marrow derived stem cell (BMSC) chondrogenesis in cartilage defect repair study (Franco et al., 2023). The tendency of exogenous mesenchymal stem cells (MSCs) to go through hypertrophic differentiation is also a long-term clinical inferiority (Wang et al., 2019a). Unexpected outcomes of neuronal MSC differentiation also remained a complicated challenge, with the appearance of de-differentiation, poor differentiation products, limited functionality, and many more as reviewed in George et al. (2019). In addition to that, genomic instability and the risk of tumour formation were among the obstacles mentioned by Golchin et al. (2021) in the differentiation of human embryonic stem cells (hESC). Whereas (Hoang et al., 2022) highlighted immunogenicity and post-administration concerns of MSCs, as well as laborious and time-consuming protocols for human induced pluripotent stem cell (hiPSC)-derived retinal pigment epithelium (RPE) differentiation. Other limitations are inadequate spontaneous migration of endogenous MSC towards impaired bone site for cartilage regeneration (Wang et al., 2019a), and loss of implanted exogenous MSCs in bone tissue regeneration (Wang et al., 2023). However, there are other hurdles to guide stem cell differentiation that are not mentioned here, which, together, greatly forced scientists to look for solutions so that stem cell-based research and therapies continue to be the future promise.

**FIGURE 1:**
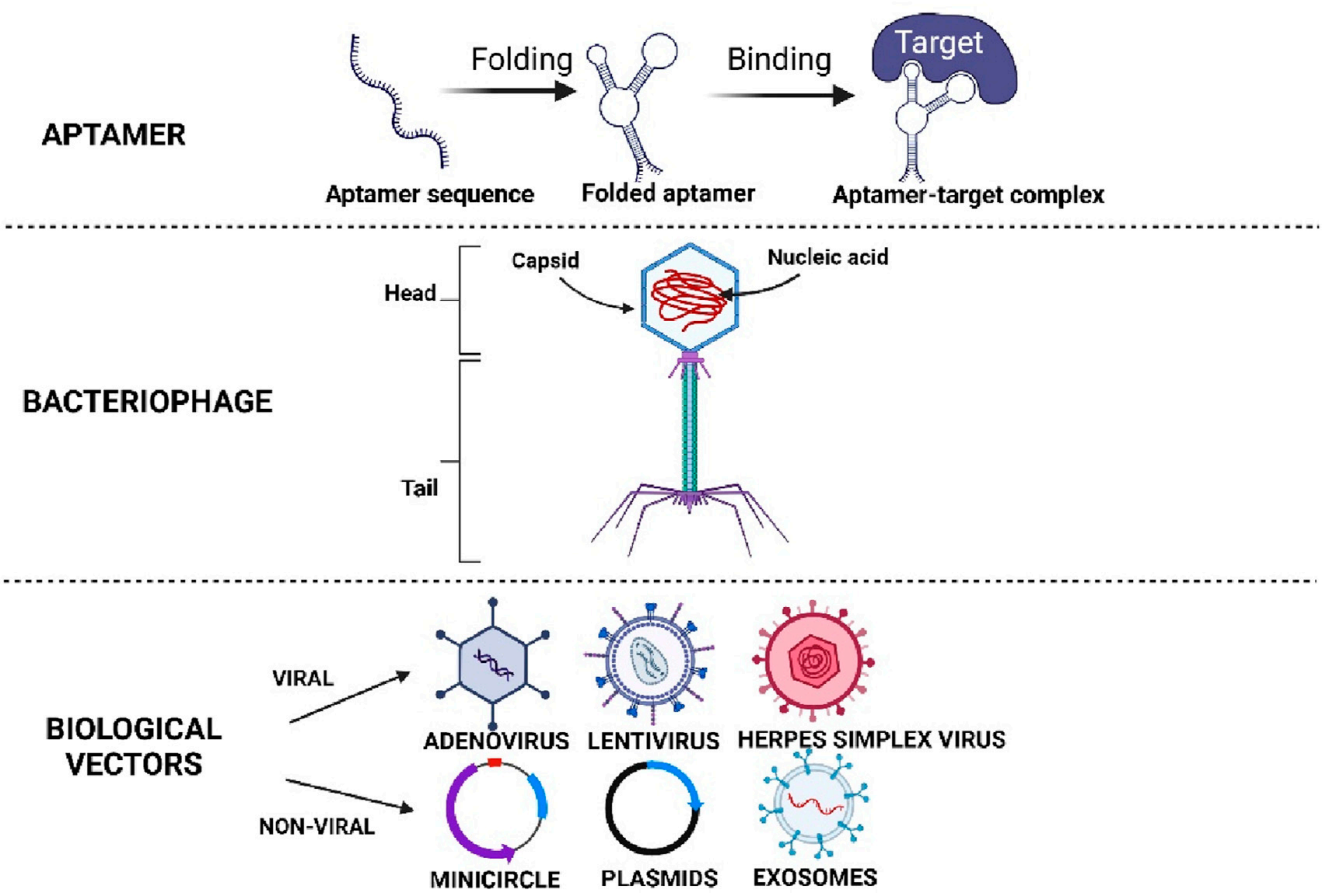
Illustrates the different types of designing molecules. Created with BioRender.com.

To overcome these limitations, many methods and technologies were explored. This includes the use of design molecules such as aptamer, phage, biological vector, and 3-dimensional (3D) biomaterial. Design molecules are molecules that are designed to modulate desired functions. Aptamer, phage, biological vector, and 3D biomaterial are some examples of design molecules used in recent tissue engineering and therapeutic studies. They are designed to specifically recognise their target cells or molecules, and perform their intended functions such as recruiting cells, displaying ligands, delivering specific genes, mimicking niches, or providing cues to direct stem cell differentiation thus, promoting cell or tissue regeneration. These strategies have proven to be the most effective approaches for directing stem cell differentiation, depending on the application and non-specific response (Jang et al., 2020; Xing et al., 2019). Biochemical factors such as small molecules and growth factors generally have a short half-life, requiring greater dosage or repeated administrations, and may evoke undesired multiple lineage differentiation (Halim et al., 2020). Consequently, effective and specific designed delivery systems are needed to enable controlled and prolonged release of these biochemical factors, especially for *in vivo* studies (Oliveira et al., 2021). For these reasons, design molecules like aptamer, phage and biological vector as illustrated in Figure 1 are being developed.

Aptamer and phage can specifically target and recruit more endogenous MSCs at the site of injury, as well as regulating biochemical factors or pathways involved in promoting tissue regeneration (Wan et al., 2019; Wang et al., 2019a; Wang et al., 2023). In this way, issues like insufficient migration and unspecific differentiation of endogenous or exogenous MSCs can be resolved through their high specificity and binding ability toward the targeted cells or molecules. On the other hand, biological vectors that include both viral and non-viral vectors are excellent tools for gene delivery (Powell et al., 2015). In addition to allowing the delivery of specific genes to targeted cells, they can also be engineered to enhance target specificity and transgene expression for short-term or permanent long-term expression in targeted cells or tissues (Lundstrom, 2018; Ghosh et al., 2020). Therefore, non-specific reactions can be reduced and safe use in directing stem cell differentiation could be better ensured. In this review, we focus on the discussion of aptamers, phage, and biological vector as design molecules, as well as their roles in directing stem cell differentiation. Hence, the 3D biomaterial and other molecules are excluded from this review.

## 2 Aptamer

Aptamer is a short, 25–80 bases long single-stranded oligonucleotide sequence that resembles monoclonal antibody (Ni et al., 2021). It can fold into tertiary structures and bind with high specificity and affinity to the targets (Figure 1) (Ni et al., 2021). There are two main types of aptamers, nucleic acid aptamers and peptide aptamers. Nucleic acid aptamers consist of nucleotide sequences that form complex secondary or tertiary structures that bind to targets using their entire sequence. However, peptide aptamers are amino acid sequences with a short peptide region embedded within a scaffold protein that acts as the backbone (Söylemez et al., 2019). The peptide region is involved in binding with the target protein while the binding specificity and affinity are enhanced by the scaffold conformation (Söylemez et al., 2019).

When mentioning aptamers however, it usually refers to nucleic acid aptamers instead of peptide aptamers, unless specifically stated. Also, in terms of directing stem cell differentiation, nucleic acid aptamers are more widely used compared to peptide aptamers which are more applicable in other research fields. Leaving that aside, nucleic acid aptamers can be further classified into DNA and RNA aptamers, whereby DNA aptamers are preferred since they are chemically more stable and do not require reverse transcription steps when developing RNA aptamers (Söylemez et al., 2019; Acquah et al., 2020). Nevertheless, the interesting characteristics possessed by aptamers have attracted scientists to employ them for various research applications in many ways.

**FIGURE 2:**
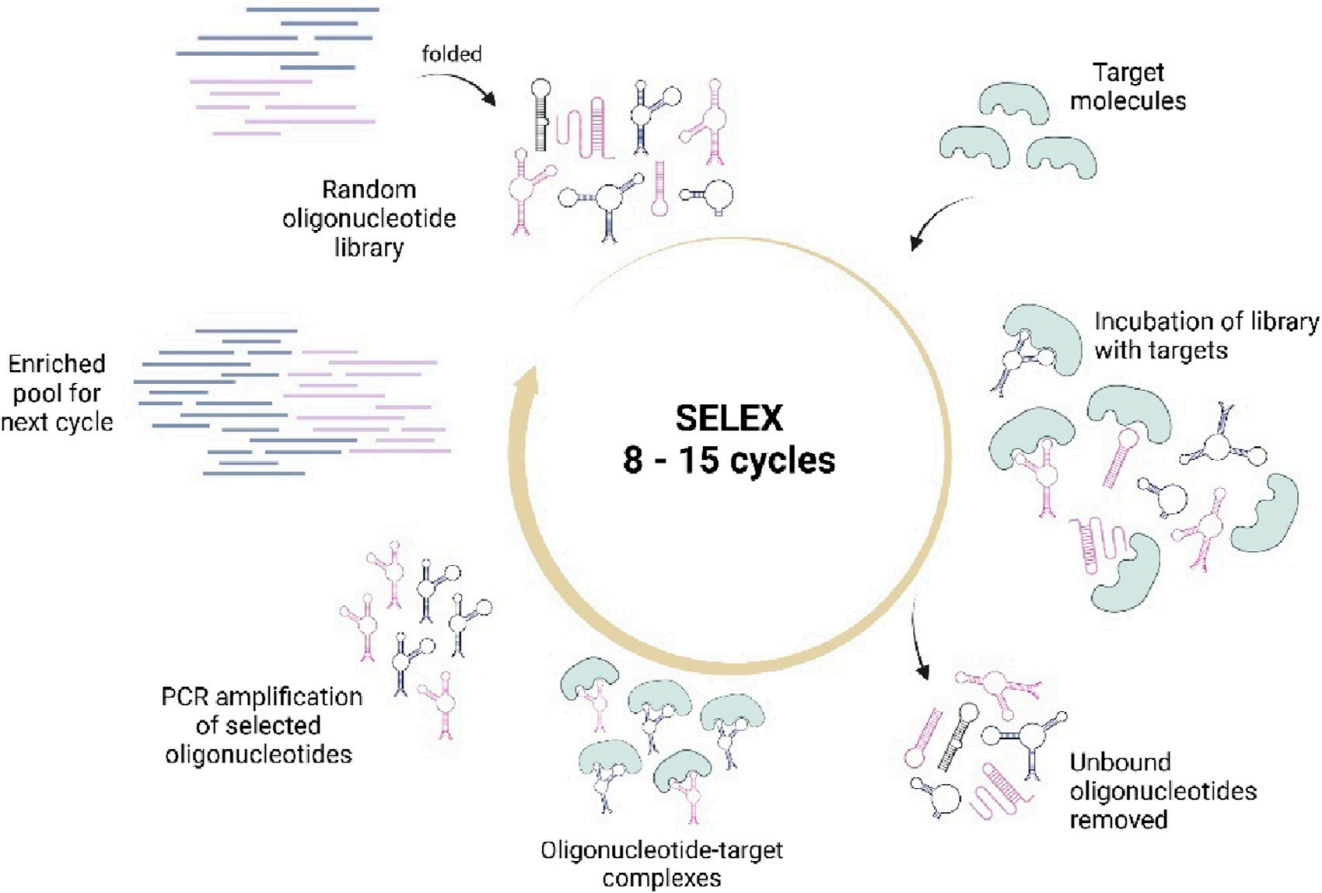
Illustrates the conventional SELEX process that involves the synthesis of a random oligonucleotide library, incubation with targets, selection of target-bound sequences, and PCR amplification for pool enrichment. The cycles are repeated until high quality aptamer sequences are obtained. Created with BioRender.com.

The aptamers are usually developed through a conventional method known as SELEX (Systematic Evolution of Ligands by EXponential Enrichment). It is an *in vitro* selection process that isolates aptamers with high affinity and specificity toward their targets (Söylemez et al., 2019; Ni et al., 2021). To simplify, SELEX involves the synthesis of a random oligonucleotide library and introduced with target molecules to obtain fragments that could specifically bind to the targets (Figure 2). This selection process is usually carried out in 8–15 rounds to obtain high affinity aptamers (Söylemez et al., 2019). However, due to time-consuming and laborious process, researchers have modified it with different methodologies such as Microfluidic SELEX, High Fidelity (Hi-Fi) SELEX, Atomic Force Microscopy (AFM) SELEX, as well as incorporating the Next-Generation Sequencing (NGS), High throughput-SELEX (HTS) which have reduced the selection cycles into just about two to six cycles, along with other types of SELEX offering different benefits, as reviewed in (Bayat et al., 2018; Komarova and Kuznetsov, 2019; Acquah et al., 2020).

However, since SELEX involves polymerase chain reaction (PCR) to amplify the target-bound aptamer sequences, it poses some disadvantages in determining aptamer candidates. The success rate remains low and the overall process still consumes a great amount of time and effort (Chen et al., 2021; Lee et al., 2023). The quality of aptamer products can also be affected by PCR amplification bias and accumulated flaws in the multistep SELEX procedure (Komarova and Kuznetsov, 2019). Hence, silico approaches in designing the aptamers have been introduced and incorporated into some SELEX methods. In silico methods are computer-based (Navien et al., 2021) and involve four main steps; 1) secondary structure prediction, 2) tertiary structure optimization, 3) structural docking simulation, 4) molecular dynamics simulation, and then followed by modifications for further quality improvements (Buglak et al., 2020; Chen et al., 2021; Lee et al., 2023). Some examples of programmes used in these steps are listed in Table 1 below (Ahmad et al., 2021; Buglak et al., 2020; Chen et al., 2021; Lee et al., 2023; Navien et al., 2021). It should be noted that the tools are not limited to those listed, as there are many others available for diverse applications.

**TABLE 1:** Shows the list of several available *in silico* programmes with different purposes.

*In silico* design	Software	Description
2D structure prediction	RNAfold, Mfold, Vfold2d, CentroidFold, RNAstructure, UNAFOLD	Predict the secondary structure based on the sequence
3D structure optimisation	RNAComposer, 3dRNA, Vfold3D, SimRNA	To optimise tertiary structure through secondary structure adoption
Structural docking simulation	AutoDock, AutoDock Vina, ZDOCK, DOCK, MDockPP, Hex, GRAMM, HADDOCK	Predict the structure of the aptamer-target complex
Molecular dynamics simulation	AMBER, GROMACS, NAMD	To evaluate stability of aptamer-target complex

Besides, some studies have also used Quantitative Structure-Activity Relationship (QSAR) method for aptamer design and prediction of binding ability (Kumar and Kumar, 2020; Yu et al., 2019). Other techniques in developing aptamer are particle display and multiparametric particle display that involve fluorescence-activating cell sorting (FACS) aptamer screening, alkyne-azide chemistry-based particle display, and non-natural aptamer array (N2A2) platform for base-modified aptamers (Wu et al., 2022). On the other side, non-equilibrium capillary electrophoresis of equilibrium mixtures (NECEEM)-based non-SELEX was also developed to eliminate the repetitive PCR amplification steps in SELEX-based procedures thus, reducing the errors they posed (Bayat et al., 2018). Whereas, to increase the quality of SELEX products and aptamers efficacy, chemical modifications have been implemented and can be applied pre- or after SELEX (Bayat et al., 2018). Modifications to nucleotide bases, sugar rings, and phosphates have improved the binding affinity and total number of aptamer candidates obtained in SELEX (Bayat et al., 2018). Chemical modification is one of the most effective approaches to improve delivery of oligonucleotides. Nucleic acid backbone modification, ribosomal sugar moiety modification and nucleobase modification have all extensively used to improve the drug delivery (Roberts et al., 2020). Modification is used to improve the oligonucleoside pharmacokinetics, pharmacodynamics and biodistribution. Most of the chemical modification method have a higher binding affinity and decrease the immune stimulation (Dhuri et al., 2020). Various base modifications and the creation of mirror image aptamers, or Spiegelmers, have facilitated the avoidance of nuclease degradation, while the composition of aptamers with bulky moieties helped to minimize the quick elimination of renal filtration (Bayat et al., 2018; Ni et al., 2021). Spiegelmers also known as synthetically prepared oligonucleotide with L-configuration, are not only highly selective and ligand-like, but also bio structural due to their resistance to enzyme in biological fluids (Rana et al., 2021). Although seems favorable, there are issues arise when dealing with chemical modifications, as unnatural nucleotides, and addition of bulky moieties like PEG (polyethylene glycol) may result in chemical toxicity and immune reactions risks respectively (Ni et al., 2021).

Whilst all these strategies are available, producing good quality aptamers are largely influenced by the aptamer sequence design since their binding specificity and affinity are target-dependent. So, knowing the targets (cells or molecules) and the way they work well will assist in good aptamer sequence creations. Furthermore, as aptamer synthesis generally consist of many steps, conducting each step, especially the initial oligonucleotide library design (Bayat et al., 2018) with great care and precision would certainly reduce the accumulation of faults thus, generating more and better aptamer products. The incorporation of suitable modifications as well would guide in the quality enhancement.

### 2.1 Advantages and disadvantages of aptamer

In terms of the main role of antibodies, the use of aptamers in many studies has been preferred due to the benefits they offer, especially the ease of production and its cost-effectiveness. Aptamers can be produced in large quantities with high accuracy, low batch-to-batch variation, and easily modified (Söylemez et al., 2019; Zhang, Lai, et al., 2019a). They are also stable in high temperatures and can be used in a wide range of assay conditions. Furthermore, they have low molecular weight and showed low immunogenicity and toxicity compared to antibodies (Sun et al., 2021; Zhang et al., 2019a). The high affinity and specificity towards target molecules (Zhang et al., 2019b), plus rapid tissue penetration and cell internalization of aptamers are some of the reasons that made them especially attractive in research studies (Wan et al., 2019). However, there are certain disadvantages to aptamers. One of them are the susceptibility towards nuclease degradation which contributes to low stability in biological fluids (Yu et al., 2016). Limited nucleic acid library which are essentially made up of four canonical nucleotides have also produced limited aptamers available for selection process (Söylemez et al., 2019). Other concerns about aptamers are that they appeared to have difficulties crossing the cell membrane especially for *in vivo* (Söylemez et al., 2019), as well as short half-lives (Yu et al., 2016).

### 2.2 Application of aptamer in directing stem cell differentiation

With all of the abilities of aptamers discussed, they have been applied to direct stem cell differentiation in many ways. In a study carried out by (Li et al., 2022), aptamer specific to MSCs was used in the repair of meniscus tears of knee joint. The aptamers Apt19S were chemically conjugated into a scaffold along with loading of growth factors that were then implanted into the site of injury. The growth factors involved were connective tissue growth factor (CTGF), which induces profibrogenic differentiation, and transforming growth factor-β3 (TGF-β3) that remodels the fibrous matrix into fibrocartilaginous matrix. Apt19S that specifically bind to MSCs are responsible for targeting and recruiting endogenous MSCs hence, maximizing the proliferation and differentiation effects of CTGF and TGF-β3 in guiding meniscal regeneration.

To achieve these dynamic effects, the functional scaffold was designed to sequentially release the aptamers and growth factors, where Apt19S was first released immediately to capture the MSCs. Bioactive scaffold used for meniscal tissue engineering primarily provides interconnected complex microchannels for endogenous cell adhesion and infiltration, as well as providing optimal physical strength to support biomechanical support. The optimum scaffolds pore sizes are between 60 and 220um, considering similar component compared to collagen scaffolds which meet the requirement of cell growth, proliferation and differentiation of cells in both dry and hydrating conditions (Li et al., 2021a). Aptamer Apt19S specifically recognizes and binds to MSCs, thereby promoting osteochondral when immobilized in scaffolds (Wang et al., 2019b). Sequential release of CTGF and TGF-β3 promotes MSC differentiation to fibrochondrocytes *in vitro* and *in vivo* (Lee et al., 2014). Therefore, CTGF and TGF-β3 are incorporated in PLGA NPs and MPs. *In vitro* growth factor release profiles shows that CTGF is released more rapidly than TGF-β3, which may need to induce heterogenous MSC differentiation. *In vitro* result shows that introduction of two growth factors could significantly improve chondrogenic and fibro-chondrogenic differentiation after 7 days of culture (Li et al., 2022).” Subsequently, CTGF was released rapidly while TGF-β3 being steadily released for meniscal fibrocartilaginous regeneration. The *in vitro* results showed enhanced cell mobilization, proliferation, and fibrocartilaginous differentiation whereas for *in vivo*, there were elevation of neo meniscal formation (Li et al., 2022).

On the other hand, RNA aptamers, RBM-007 were used in treating a genetic disease, achondroplasia (ACH) which causes dwarfism in humans (Kimura et al., 2021). ACH occurs due to a mutation in the fibroblast growth factor receptor 3 (FGFR3) that causes abnormal activity of certain signaling pathways detrimental in chondrocyte proliferation and differentiation for bone growth. FGFR3 however, is activated by fibroblast growth factors (FGFs) hence, RBM-007 that specifically binds to one of its corresponding ligands, FGF2 was used in this study to prevent the activation of FGFR3. The results indeed depicted inhibition of FGFR3 signalling, and RBM-007 also repaired defective chondrocyte differentiation in tibial organ culture, *in vitro* and *in vivo* ACH chondrocyte differentiation, as well as skeletal defects in the ACH mice model, suggesting that RBM-007 is potentially useful in ACH and other FGFR3-related skeletal dysplasia therapy (Kimura et al., 2021). Among these two studies, other of aptamers on the application studies are included and can be referred to in Table 2 below.

**TABLE 2:** Shows examples of aptamer applications in the direction of stem cell differentiation.

Disease	Aptamer	Application	References
Meniscus tear	Apt19S	Apt19S-mediated MSC-specific with growth factors developed as scaffold promotes *in vitro* cell migration, proliferation, and fibrocartilaginous differentiation, guiding *in vivo* meniscal regeneration	Li et al. (2022)
Achondroplasia (ACH)	RBM-007	The RNA aptamer, RBM-007 inhibited FGFR3 signaling through FGF2 ligand neutralisation and restored defective chondrocyte differentiation *in vitro* and *in vivo*, as well as skeletal defects in ACH mice	Kimura et al. (2021)
Osteoporosis	BMSC-specific aptamer	The surface of bone marrow stromal cell (ST) derived exosomes (STExo) surface is conjugated with a BMSC-specific aptamer which helps to deliver STExo to BMSC within targeted bone marrow and promote *in vitro* osteogenesis and *in vivo* bone regeneration	Luo et al. (2019)
Articular cartilage tissue regeneration	Apt19S	Apt19S used as part of injectable hydrogel scaffold along with small bioactive molecule (Kartogenin) were released sequentially and then promoted endogenous MSC recruitment and cartilage tissue regeneration in rabbit full-thickness cartilage defect model	Dai et al. (2023)
Bone defects	Apt19S	Apt19S sequentially released from scaffold which aids *in vitro* cell migration and proliferation, mobilisation and recruitment of *in vivo* host BMSCs to defective sites in a large-scale bone defect rat model, enabling efficient osteogenic differentiation	Sun et al. (2021)
Bone regeneration	3R02 bivalent aptamer	Bivalent 3R02 aptamer specific to vascular endothelial growth factor (VEGF) conjugated to the surface of hydroxyapatite (HA). Apt-HA showed greater ability to capture VEGF that promotes *in vitro* cell proliferation, and *in vivo* angiogenesis and bone regeneration in tibial metaphyseal defects rabbit model	Son et al. (2019)
Bone defects	HM69 and HM69-NAB	Produced novel aptamer HM69 and HM69-functionalised nanoparticles NAB (Nano-Aptamer Ball) effectively recruits MSCs both *in vitro* and *in vivo* which enhances bone defects recovery in rat model	Wang et al. (2019a)

## 3 Bacteriophage

Bacteriophage, or phage, is a human-safe bacteria-infecting virus made up of protein capsid as the outer structure, while containing its genetic materials (Figure 1) (Cao et al., 2019). There are two lifecycles, the lytic and lysogenic lifecycles, which the phage could undergo. For lytic lifecycle, the host cell’s machinery system will be taken over upon the injection of the phage’s genome to produce more of itself. The lysogenic lifecycle only involves the integration of the phage genome into the host genome and will remain dormant and replicated along with the host during cell division (Monteiro et al., 2019). These distinctive lifecycle modes consequently classify phage into different types, the lytic, temperate, and non-lytic phage (Cao et al., 2019; Monteiro et al., 2019). Basically, lytic and nonlytic phages strictly undergo lytic life cycle and lysogenic lifecycle respectively, while temperate phages carry out lysogenic lifecycle and could switch into lytic lifecycle when the host cell is put under environmental stress (Cao et al., 2019; Monteiro et al., 2019).

Exploiting the intriguing nature of phage, scientists have been experimenting with phage for various research purposes such as delivering or expressing desired genes, displaying desired coat protein, targeting specific bacteria or cells, and many others, which mostly were made possible through advancements of genetic engineering. In general, different types of phages are utilized according to their natural behaviours. Instinctively, a lytic phage will be used when its lytic activity is needed. Otherwise, non-lytic phage will be preferred. Temperate phages have been previously less used since they pose the risk of unexpected lifecycle switching. But advances in technologies have now made researchers explore ways to utilize them, by eliminating lysogenic-contributing genes that convert them into lytic phage and omit concerns of possible horizontal gene transfer that can cause bacterial resistance or superinfection immunity (Monteiro et al., 2019; Peng and Chen, 2021). Likewise, when bactericidal effect and its accompanied release of endotoxin are undesired, lytic genes can be preferably inhibited (Monteiro et al., 2019; Peng and Chen, 2021). Therefore, scientists have the opportunity to investigate the potential use of all types of phages, allowing them to be applied in various studies.

**FIGURE 3:**
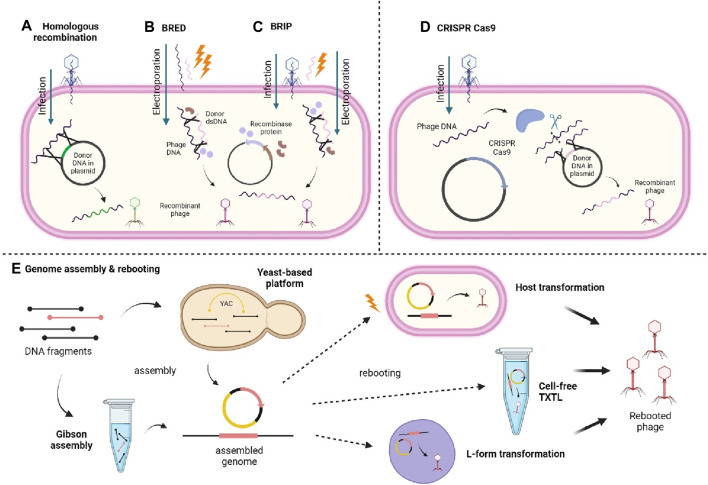
Illustrates different methods of synthesising recombinant phage. **(A)** Homologous recombination between the injected phage genome and the donor DNA carried in a plasmid. **(B)** BRED involves the electroporation of the phage genome and donor dsDNA into the cell carrying a recombinase-encoding plasmid. **(C)** BRIP works similarly to BRED except the phage genome is injected by the phage itself instead of being electroporated. **(D)** CRISPR Cas9 involves use of CRISPR Cas9 system to nick the phage DNA and allows recombination with donor DNA contained within a plasmid. **(E)** Genome assembly and rebooting involves the assembly of DNA fragments through yeast-based platform or Gibson assembly and then rebooted through transformation into host or L-form bacterial cells, or using cell-free TXTL system. Created with BioRender.com.

Many methods have been used to develop and designing phage with desirable features. The phage genome can be modified through genetic engineering methods (Figure 3) such as homologous recombination between the phage and donor DNA, producing new recombinant phages (Monteiro et al., 2019; Lenneman et al., 2021; Mahler et al., 2023). Also, bacteriophage recombineering of electroporated DNA (BRED) method has improved homologous recombination through electroporation of phage DNA and dsDNA containing desired modification into host cell carrying recombinase protein-encoding plasmid hence, increasing the rate of recombination (Monteiro et al., 2019; Mahler et al., 2023). Another variant called bacteriophage recombineering with infectious particles (BRIP) instead, involves only the electroporation of the dsDNA, while the phage genome is delivered through usual phage infection (Mahler et al., 2023). Furthermore, fragments of synthetically produced phage genomes can also be assembled and subjected to genome rebooting to produce functional phages. The synthetic fragments can be assembled through yeast-based platform and Gibson assembly (Lenneman et al., 2021; Mahler et al., 2023). The yeast-based platform involves homologous recombination between fragments and the yeast artificial chromosome (YAC), creating YAC-phage DNA, while Gibson assembly implies an *in vitro* enzymatic assembly of synthetic genomes (Monteiro et al., 2019; Lenneman et al., 2021; Mahler et al., 2023). The resulting fully assembled genomes from either method will then be rebooted through transformation into host or L-form bacterial cells for recovery of functional phages, or using cell-free transcription-translation (TXTL) systems (Monteiro et al., 2019; Lenneman et al., 2021; Mahler et al., 2023).

Apart from that, clustered regularly interspaced short palindromic repeats-CRISPR associated protein (CRISPR-Cas) genome editing has also been employed in phage engineering and there are many CRISPR-Cas systems that can be used (Mahler et al., 2023). For example, CRISPR-Cas9 nuclease is used to cleave the injected phage genome to allow recombination of the phage genome and donor DNA, and the recovery of engineered phages (Monteiro et al., 2019). It should be brought to attention though that the use of CRISPR-Cas systems is, however, restricted to CRISPR-Cas encoding bacteria (Mahler et al., 2023). In addition to phage engineering, many researchers have also modified the capsid of the phage protein to display functional proteins (phage display) by adding amino groups, decoration proteins, and chemical modifications by bio conjugating functional groups (Carmody et al., 2021; Peng and Chen, 2021). Chemical modifications are generally less complicated than genetic engineering, yet there might be chemical changes induced that could interfere with the phage’s functions (Peng and Chen, 2021).

Despite the various methods used to develop and design phages with favorable features, achieving phages with desired functionality essentially depends on the type of phage used and the molecules or cells to be displayed or targeted by the modified phage. Hence, the modification of the phage sequences or genes needs to be well considered and accurate in ensuring the required specificity of phages for their targets. As mentioned before, different phages work differently and so the suitable phage must be carefully chosen to meet the targeted purpose in studies. Ergo, understanding the phage itself as well as the target molecules or cells are detrimental in achieving high quality phage with expected functionality and reliability.

### 3.1 Advantages and disadvantages of bacteriophage

The phages present several advantages that made them widely used. They can be easily produced in large quantities due to their error-free replication nature and the specificity of infection thus, reducing the manufacturing cost (Cao et al., 2019; Shrestha and Yoo, 2019). The ability to display not only a single peptide also made it easier for researchers to obtain multiple peptides display with concerted functionality (Cao et al., 2019; Shrestha and Yoo, 2019). Other advantages include being stable under physiological conditions so that its infectivity can be retained in intravenous (IV) injection and in the circulation *in vivo*, as well as easily assembled or introduced into scaffolds (Cao et al., 2019). In addition, the natural existence of phages in the human body denotes their safe *in vivo* use for humans. Still, researchers face challenges when working with them. Some studies reported a possible cause of the range of immune responses when administering filamentous phage (Henry et al., 2015; Cao et al., 2019). This is connected with the uncertain safety problems when using phage, therefore requiring more preclinical and clinical studies to secure its safety and efficacy, especially for human administration (Caflisch et al., 2019; Nale and Clokie, 2021). In addition to that, phages can also be quickly cleared out of the *in vivo* system shortly after implantation due to the short clearance rate (Podlacha et al., 2021). Plus, the range of phage that can be utilized could be limited to known and characterized phage hence, demanding more exploration and understanding of other potential phages (Lemire et al., 2018).

### 3.2 Application of phage in directing stem cell differentiation

As discussed above, the characteristics of the phages and the desired modifications applied allow them to be used in studies involving directing stem cell differentiation (Cao et al., 2019). In the treatment of ischemic cardiovascular diseases, Jang et al., 2020 had genetically engineered M13 phages to increase the function and graft capacity of human cardiac progenitor cells (hCPC) that are being disrupted by loss of blood vessels and endothelial cells. The M13 phages were engineered to display two functional peptides, RGD and SDKP on PIII site to enhance hCPCs cell adhesion ability, and on PVII site for anti-fibrotic and angiogenic effects, respectively. For *in vitro* observation, hCPCs were primed with M13 engineered peptide carriers and the angiogenic potential was shown to improve through elevated wound healing and tube formation capacity. Subsequently, hCPCs with M13 peptide carriers were transplanted into the ischemic region of the mice model and the results demonstrated a higher graft rate, cell survival, cardiac function, and angiogenesis. These data proposed the potential use of hCPCs pre-treated with engineered M13 peptides-displaying phages in ischemic cardiovascular disease therapy although they had also found the absence of effects on morphological changes, proliferation, and differentiation capacity of *in vitro* pre-treated hCPCs (Jang et al., 2020).

Furthermore, filamentous phages were utilized in a study involving stroke therapy (Liu et al., 2022). Infarcted brain tissues caused by stroke lead to cavity formation that hampers brain tissue due to angiogenesis and neurogenesis depletion in the cavity (Liu et al., 2022). Hence, filamentous phages were genetically engineered to display high copies of RGD peptides (R-phage) and coated electrostatically on biocompatible injectable silk protein microparticles. The R-phage-coated microparticles were then seeded with neural stem cells (NSCs) and injected into stroke site of rat brains. From the results, the R-phage in the microparticles had increased NSCs adhesion, proliferation, and infiltration in the stroke cavity. It also promoted angiogenesis through revascularization in the brain and impelled neural differentiation of the NSCs on the microparticles, thus facilitating repair of impaired brain tissue and restore motor control of the stroke rat models (Liu et al., 2022). Apart from these examples, other applications of phage in directing stem cell differentiation and tissue regeneration are also included and can be referred to in the table below (Table 3).

**TABLE 3:** Shows examples of phage applications in directing stem cell differentiation.

Disease	Phage	Application	References
Ischemic cardiovascular diseases	M13 phage	The M13 phage (peptide carrier), genetically engineered the coat protein to express two functional peptides (RGD & SDKP). Human cardiac progenitor cells, hCPCs pretreated with the engineered M13 phage *in vitro* showed improved angiogenic capacity and better cell survival and angiogenesis *in vivo* in the ischemic region where the pre-treated hCPCs were grafted	Jang et al. (2020)
Peripheral nerve injury	M13 phage	M13 phage genetically engineered to display high RGD peptide density (R-phage). The R-phages were integrated with biocompatible injectable fibrin gel, creating an R-phage/FG hydrogel and seeded with neural stem cells (NSCs). This enhances NSC adhesion, infiltration and proliferation, induces efficient preferential differentiation of NSC, and stimulates neurogenesis in the long-gap defect of the rat sciatic nerve	Chen et al. (2023)
Stroke	Filamentous phage	The filamentous phage, genetically engineered to display high RGD peptide copies were electrostatically coated on NSC-loaded silk protein microparticles. NSC adhesion, proliferation, infiltration, and preferential differentiation were promoted, as well as stimulated angiogenesis and neurogenesis at stroke sites of rat models	Liu et al. (2022)
Dorsal root injury	Ph.D.-7 library	Phage display peptide library, Ph.D.-7 library used for biopanning, identifying small peptides that target FGFR2, important for nerve regeneration and functional recovery. Peptide CH02 displayed through phage biopanning has the best binding ability. CH02 sustained neuron survival and increased axon growth *in vitro* and promoted nerve regeneration and sensory motor behavioral recovery in dorsal root injury through FGFR signaling activation	Zhao et al. (2021a)
Ischemic diseases	M13 phage (YSY184)	M13 phage genetically engineered into RGD-displaying phage (R-phage) were seeded with endothelial progenitor cells (EPCs) and transplanted into mouse hindlimb ischemia model. The R-phage mimicked extracellular matrix (ECM) niches, presenting biochemical, topographical, elastic, and mechanical cues when seeded on the hydrogel. Transplanted EPCs survival and differentiation were elevated, as well as angiogenesis at the ischemic sites	Shrestha and Yoo (2019)

## 4 Biological vectors

During the past 20 years, gene transfer to stem cells has led to optimism that gene therapy approaches can provide long-lasting therapeutic benefits. Numerous research papers have advanced our knowledge of individual stem cell behaviour in various tissue environments. Genetic modification has played an important role in cellular biology studies that seek to elucidate cellular mechanisms and pathological processes (Reiser et al., 2005). The increased understanding of biological signaling pathways and networks has led to the emergence of a completely new area of synthetic biology where single or multiple gene transfer to cells, assigning new functions, and potentially affecting the entire metabolism of multicellular organisms (Vargas et al., 2016). Biological vectors consist of viral vectors and non-viral vectors that are used to deliver genetic materials by infecting the cells (Karami et al., 2023).

Viruses have been developed to be very efficient in providing nucleic acids to certain types of cells while eliminating immunosuppression by the infected host. These characteristics make viruses an ideal vector for gene therapy (Robbins and Ghivizzani, 1998). Gene therapy involves the delivery of specific genetic materials (DNA or RNA) via a carrier called a “transmission vector” that allows foreign genetic material to enter target cells. Viral vectors are composed of retroviruses, adenoviruses, lentiviruses, and adeno-associated viruses. Viral vectors cover a wide range of applications, including short-term expression delivery vehicles and long-term expression delivery vehicles. Both RNA and DNA viruses are represented, with a single- or double-stranded genome (Lundstrom, 2018). Nonviral vectors are composed of exosomes, piggyBac transposons, mRNAs, and plasmid DNAs.

### 4.1 Viral vectors

#### 4.1.1 Retrovirus

Retroviruses are classified into seven generations (α-, β-, γ-, δ- and ε-Retroviridae, Spumaviridae and Lentiviridae (Poletti and Mavilio, 2021a). They are RNA-based viruses that are converted into double-stranded DNA molecules and stabilized in the host genome through pre-integration complex (PIC) and integrase protein (IN). Once integrated, provirus consists of two long terminal repeats (LTRs) and sequences for reverse transcription and packaging. Simple retroviruses, such as γ-retroviruses, contain only four basic genes, gag, pro, pol, and env. These genes are necessary for the viral particle production, assembly, and post-entry processing that eventually lead to proviral integration (Chameettachal et al., 2023). Gag encodes a precursor protein processed into the mature MA (matrix), CA (capsid), and NC (nucleocapsid) proteins. Pol encodes the IN and reverse transcriptase (RT) enzymes. Pro encodes the protease (PR) that processes the gag precursor. Env encodes the surface glycoprotein (SU) and transmembrane (TM) domains of the viral envelope that associate with specific cellular receptors and mediates fusion with the cell membrane (Koo et al., 2014).

As for the method of designing, retroviral vectors can be employed to stimulate the production of a particular protein in transgenic cells. The most basic approach involves the use of a promoter in a retroviral long terminal repeat (LTR) to regulate the expression of the cDNA encoding the desired protein. Alterations are made to ensure tissue-specific expression or inducibility of the gene of interest. Retroviral sequences present in the inserted gene do not significantly influence gene expressions (Adam et al., 1995). An internal promoter can be used to express a single coding region, allowing greater flexibility in the selection of promoters. These expression strategies are most effective when the gene is a selective marker such as hypoxanthine-guanine phosphoribosyl transferase (hprt) which facilitates the selection of vector transduced cells (Miller et al., 1983). The vector can be co-transfected with a non-selectable marker on a separate plasmid to introduce it into packaging cells, avoiding potential toxicity and antigenicity. However, this approach is more challenging to generate the cells with high-titer vector stock when the inserted gene is non-selectable (Coffin et al., 1997).

The broad tropism of the lentiviral vector makes it more suitable. LVs are widely used in the reprogramming of a wide variety of somatic cells, including mice, rats and humans (Al Abbar et al., 2020). The source of the most widely used lentivirus is HIV. Lentiviruses have a higher cloning capacity and higher infection efficiency than retroviruses. Unlike MMLV, lentiviruses can replicate in dividing and non-dividing cells (Thanaskody et al., 2022). Both lentiviruses and retroviral vectors carry a high risk of insertion mutation during transfection because of their genomic integration. It is well known that lentiviral is better at integrating viral vectors than retroviral.

Because LVs integrate into actively transcribed genes, their integration pattern is determined by the specific transcriptional program of target cells (Poletti and Mavilio, 2021b). At the host genome level, LV pre-integration complex (PIC) is primarily bound to the final integration site by the ubiquitous chromatin-associated LEDGF/p75 protein and transcribed gene body regions associated with specific chromatin signatures (H4K20me1, H3K36me3, H2BK5me1, and H3K27me1) (Wang et al., 2007). This is in sharp contrast to the integration characteristics of γ-retroviral vectors, which favor active transcriptional regulatory elements (enhancers and promoters) enriched with markers H3K4me1, H3K4me3, and H3K27ac (Cattoglio et al., 2010). These priorities are determined by the different properties of the chromatin factors that bind to the γ-retroviral PIC and favor the deregulated destruction of host cell genes upon vector integration (Sharma et al., 2013).

In a study, researchers found that lentiviruses did not cause a higher tumor incidence or an earlier onset of tumors in mice compared to the use of retroviral vectors in mice (Montini et al., 2006). Lentiviral vectors can overcome the shortcomings of retroviruses. Due to their evolution from complicated retroviruses lentiviruses have several benefits over simple retroviruses: Higher virion stability Higher titer Reduced insertional mutation rate (Dufait et al., 2012).

**FIGURE 4:**
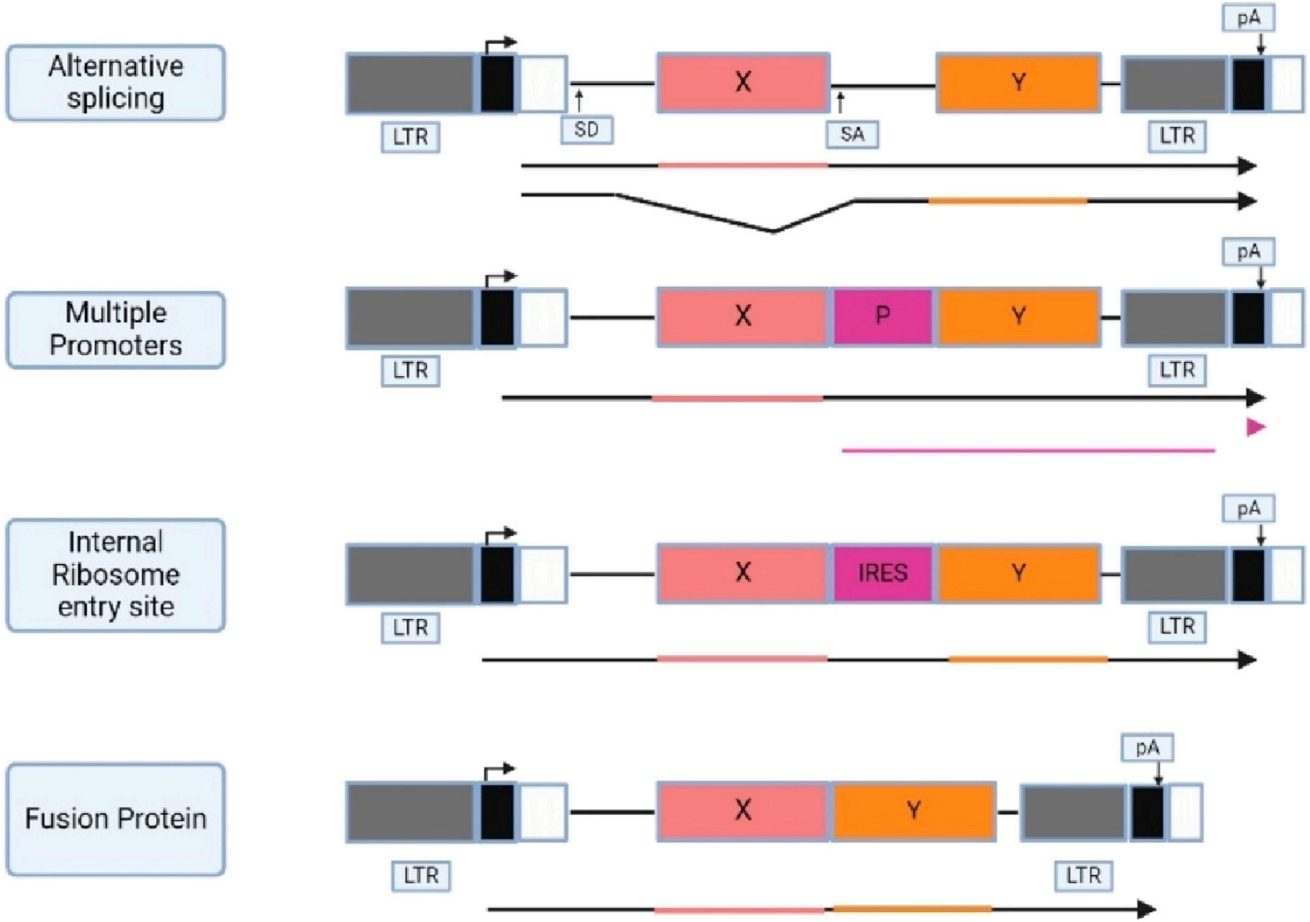
Illustrates different approaches used for the development of retroviral vector. (X and Y) protein coding regions; (P) promoter; (SD and SA) splice donor and acceptor; (IRES) internal ribosome entry site; (pA) polyadenylation signal with direction indicated by an arrow directly above. The promoters and the transcription direction are shown above the vector diagram, and the transcribed RNAs are shown below the diagram. Created with BioRender.com.

Three general approaches have been employed in the development of retroviral vector design that express multiple proteins which are using alternatively spliced messenger RNA, using promoters in LTR and internal promoters to induce transcription of cDNA, and using IRES elements to translate multiple coding regions from one mRNA. Another approach is creating a fusion protein from an open reading frame (ORF), like the fusion protein found in viruses and oncogenes. Figure 4 shows the general approaches that have been utilized for retroviral vector design.

SV(X) is a vector that uses alternative splicing to express genes from a viral LTR, including a neomycin phosphotransferase gene as an optional marker. The model for this vector is the parent virus Mo-MLV, where the Gag and Gag-Pol proteins are translated from full-length viral mRNA (Johnson et al., 2014). This strategy results in the translation of one of the proteins from the full-length vector RNA through splicing, linking the splice donor in the 5′LTR region to the spice acceptor upstream of the splice gene (Howe and Chandrashekran, 2012). The resulting RNA can be translated from the other gene product (Emery and Swanstrom, 2021). However, this approach has one disadvantage, where foreign sequences can alter the proportion of spliced and unspliced RNA or provide alternative splice genes that interfere with the production of a spliced RNA that codes for the other gene product, potentially affecting the production of encoded proteins.

Vectors with internal promoters have been extensively used for gene expression, as they allow the use of promoters other than viral LTRs (Blø et al., 2008). Retroviral vectors can contain multiple internal promoters, allowing for at least three distinct cDNAs to be expressed from their own promoter. However, selection for one gene can lead to promoter suppression, reducing the expression of another gene in the vector. It is difficult to determine whether this phenomenon is due to promoter suppression or the specific design of the experiments (Coffin et al., 1997).

The use of an internal ribosomal entry site (IRES) in retrovirus vector development is increasing, allowing cap-independent translation of a downstream gene (Fux et al., 2004). IRES sequences from picornavirus have been used successfully used in retrovirus vector. Picornavirus 2A sequences, most used in food and mouth disease aphthovirus (FMDV) are used to express two genes from a single promoter. 2A acts as a short sequence links two genes to form a polyprotein, which is post-translated into form two distinct proteins at the site of 2A. This allows for the expression of up to three distinct transgenes in a single construct (Chinnasamy et al., 2009; Howe and Chandrashekran, 2012).

#### 4.1.2 Adenoviral vectors (AdV)

Adenovirus is a non-enveloped virus primarily associated with respiratory tract infections but can also affect other organs like the brain and urinary bladder. It has an icosahedron protein capsid with 26–45 kB linear DNA genome and inverted terminal repeat sequences (ITRs) (Bulcha et al., 2021). Adenoviruses cause non-invasive respiratory tract infections in humans and animals. Some of the human serotypes are common in children, and most adults have neutralizing antibodies against them (Singh et al., 2018). Adenoviral genes can be classified into five early and five late genes. Once incorporated into target cells, adenoviruses can express the early gene E1A, E1B, E2, E3 and E4 which regulate host gene expression for the synthesis and replication (Bulcha et al., 2021; JT et al., 2022). As a highly immunogenic virus, adenoviruses fall into the level 2 risk category of National Institutes of Health (NIH) (Islam and Tom, 2022).

Endocytosis is the process of transferring a particle from a cell to inside a cell, where the ligands attached to the cell surface receptor are internalized. The fate of these ligand-ligand complexes depends on the type of vesicles. Many receptors release their ligand in the acidic medium of the endosome, which is then sorted into vesicles that are recycled to the plasma membrane. The ligands are then sorted into vesicles, which are then used to lysosomes, where enzymes degrade the release ligands (Doherty and McMahon, 2009). Endocytosis is essential for cell survival and serves as a vehicle for viruses to invade cells. Non enveloped and envelope viruses have the same basic steps and routes for the entry, starting with attachment to cell surface and leading to intracellular transfer of the viral genome (Nestić et al., 2021). Enveloped viruses can internalize in host cells through both endocytic and non-endocytic pathways, while non-enveloped viruses can only use the endocytic pathway (Nestić et al., 2021).

**FIGURE 5:**
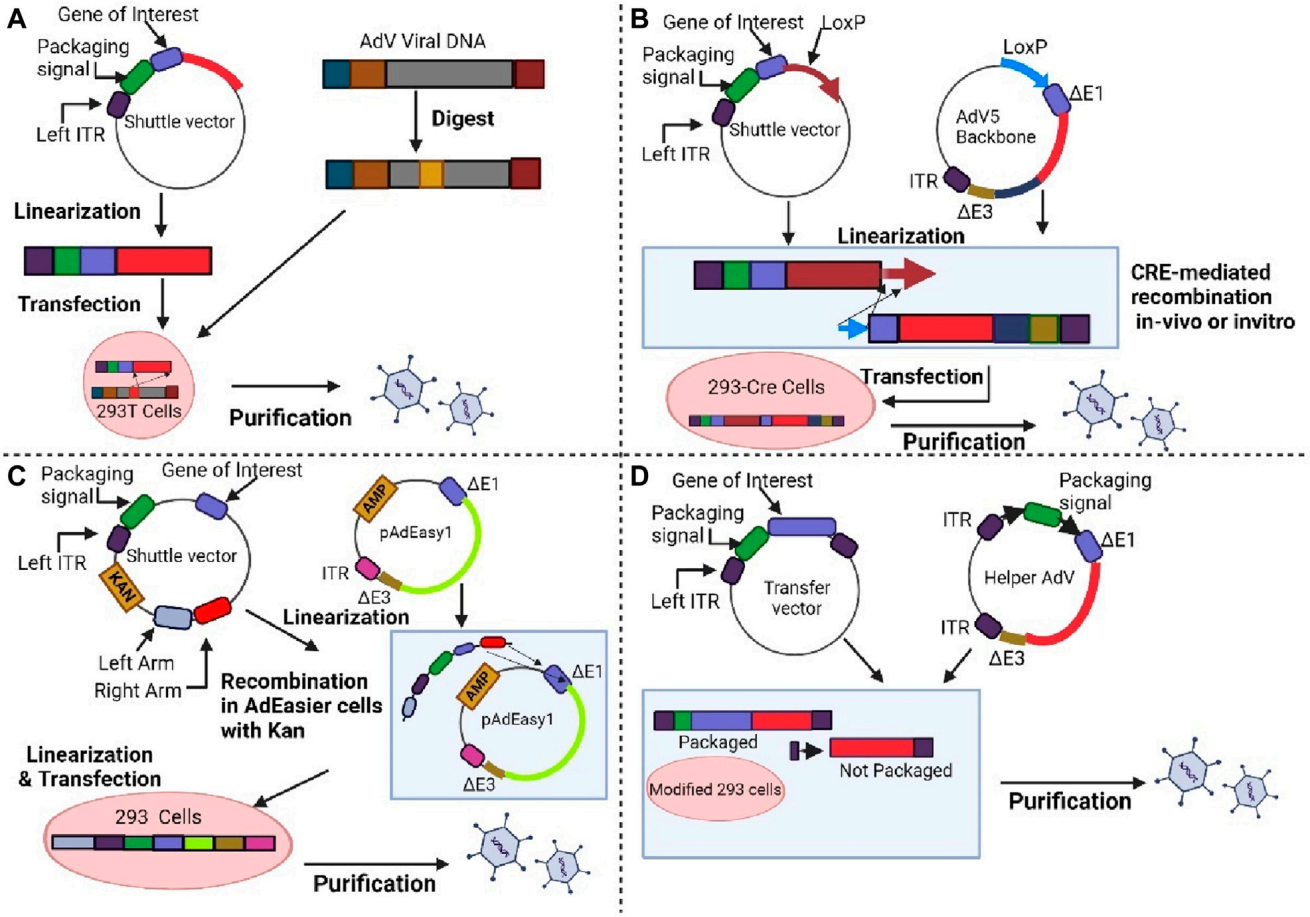
Shows the method for generating adenoviral vectors. **(A)** First generation. Adenoviral vectors are produced by the homologous recombination method. **(B)** First or second generation. The target gene in the shuttle vector and Lox-P containing adenoviral vector are incorporated by the Cre recombinase-mediated recombination method. **(C)** First or second generation. The linearized shuttle vectors are joined together with ampicillin-resistant adenoviral backbone vector, and the plasmids are selected on kanamycin. **(D)** Third generation. The transfer vector and helper adenovirus are used to generate AdV. Modified HEK-293 cells are used to produce an adenovirus vector that prevents packaging of helper adenovirus which due to deletion of packaging signal. Created with BioRender.com.

Constructing AdV vectors is still a laborious and time-consuming process. Several approaches have been developed over the years to facilitate the production and utilization of AdV vectors. For instance, the *in vitro* ligation method, homologous recombination method, cre-lox recombination method and bacterial artificial chromosome technology are further discussed by the reviewers (Syyam et al., 2022). Figure 5 shows the general approaches that have been utilized for adenoviral vector design. One of the most advantageous and effective *in vitro* gene editing approaches for the modification of adenoviruses is through the CRISPR -Cas9 system.

Ad5 is a popular adenovirus vector for gene therapy due to its clear genetic background. However, its natural cell receptor, coxsackie, is low in T cells, limiting its effectiveness in genetic alteration. Adenovirus serotype 35, part of subgroup B adenoviruses, recognizes CD46, which is highly active on human T cells (Li et al., 2021b). The efficacy of infection depends on the adenovirus entering the intracellular compartment of target cells. Recent studies have focused on tumor cell lines, but the pathway to human primary T cells remains unclear (Wang et al., 2018). A solution involves targeted editing of the adenovirus genome, replacing the Ad5 fiber gene with another serotype to enhance infection effectiveness (Zhang et al., 2016).

The CRISPR-Cas9 system is a method for targeting adenoviruses, requiring only one guide RNA (sgRNA) directed to the target sequence. SgRNA serves as a guide for the Cas9 nuclease, which binds to a specific gene sequence and induces a DNA double-strand break (Cong et al., 2013). The Ad5 fiber gene was targeted using CRISPR-Cas9 and Sequence and Ligation Independent Cloning (SLIC) technology. The adenovirus packaging system consists of a backbone plasmid with a Cre gene expression cassette and a shuttle plasmid with a loxP (Li et al., 2021b; Ng et al., 1999). Cre-mediated site-specific recombination generates an adenovirus vector through transfection of both plasmids into HEK293 that express cre recombinase.

**FIGURE 6:**
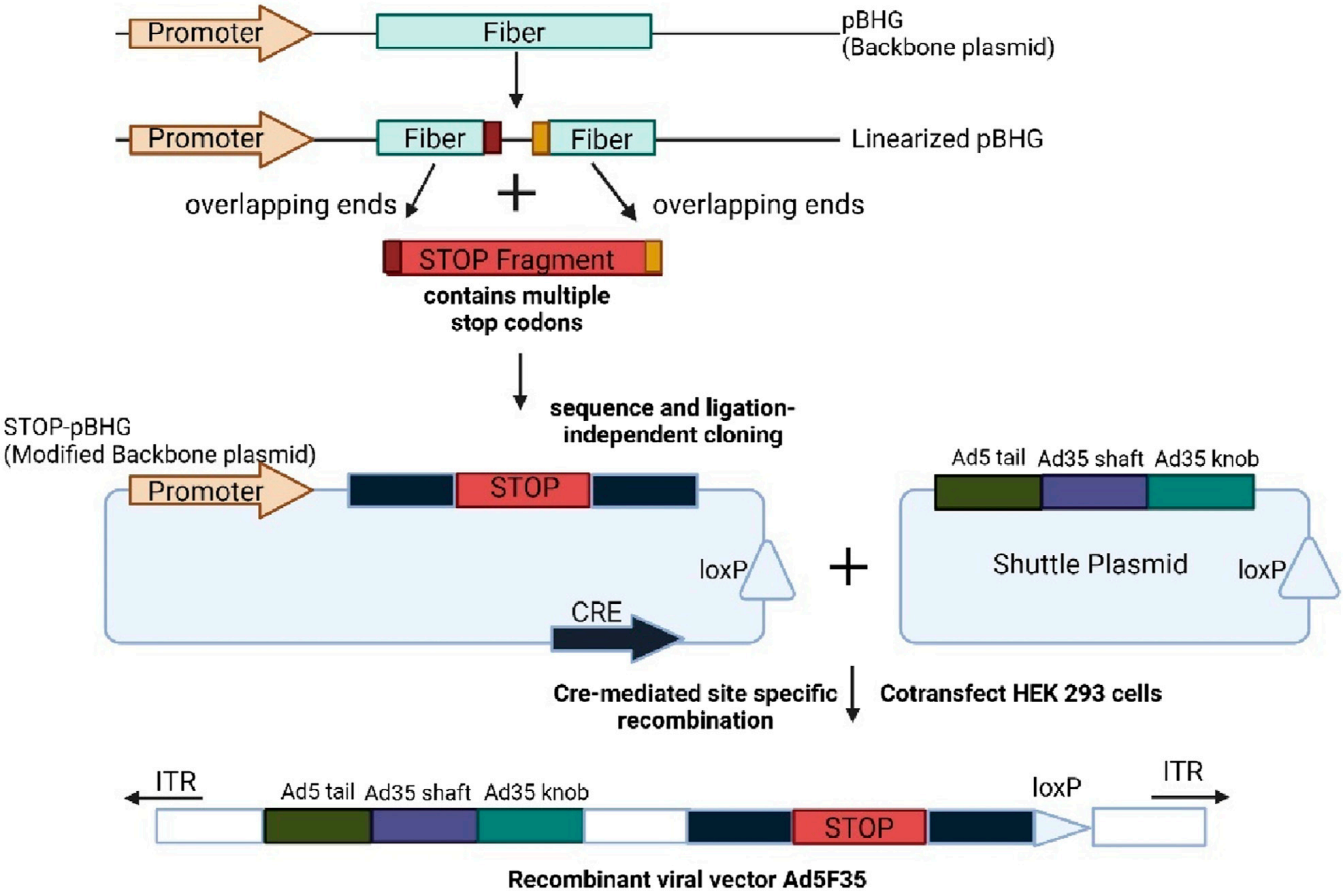
Shows the current CRISPR-CAS9 approach for targeted editing in adenoviral genome. Created with BioRender.com.

The first step involves designing and transcribe a sgRNA that targets the fiber gene before incubation with Cas9. The Cas90-sgRNA complex cleaves the adenovirus backbone plasmid *in vitro*, and the linearized plasmid is purified. The STOP fragment inactivates the genes and the Ad5 fiber gene tail fragment fused with the shaft or knob fragment of Ad35 fiber gene. The edited backbone was co-transfected with the recombinant shuttle plasmid into packaging cells to form the recombinant Ad5F53 (Ran et al., 2013). Figure 6 shows the targeted editing of the adenoviral genome using the CRISPR-Cas9 approach. This method is easy and efficient, providing the basis for the generation of viral vectors suitable for gene therapy.

#### 4.1.3 Adeno-associated viral vector (AAV)

Adeno-associated virus is a non-enveloped parvovirus. It has a 4.7 kb DNA genome with two gene sequences (rep and cap). AAV vectors have been to be safe and effective in clinical use (Maheshri et al., 2006). AAV exposure results in long term latent infection in a wide variety of dividing and non-dividing cells (Jang et al., 2011). Russell and others have shown that AAV vector genomes can mediate homologous recombination with target sequences within the cellular genome with 10^3^–10^5^ times higher efficiency than plasmid constructs (Russell and Hirata, 1998). This ability has been demonstrated to work with cells that AAV is able to effectively transduce such as fibroblasts and mesenchymal stem cells, resulting in development of novel animal models for human disease (Rogers et al., 2008). The development of novel AAV variants able to effectively infect stem cells may have implications in stem cell biology, as well as regenerative medicine.

AAV has many serotypes and variants and different AAV serotypes exhibit significant differences in the transduction efficiencies and cell and tissue tropism (Yao et al., 2023). Selection of the appropriate AAV serotype is one of the first steps in designing AAV gene delivery (Wiley et al., 2018). Method to develop AAV involves amino acid mutation, motif insertion and chemical biology. More specifically, AAV gene delivery efficiency and specificity can be enhanced by point mutations on the virus capsid. The various serine, threonine, and lysine residues in AAV2 capsid have been mutated to alanine or arginine residue (Gabriel et al., 2013). Most of these substitutions result in enhanced transduction efficiencies in HeLa cells and increased gene expression in liver of mice. In a separate study, three AAV6 mutants (F129L, Y445F, Y731F) were generated by point mutation to the capsid to produce AAV6.2FF (Lieshout et al., 2018). Point mutation to AAV capsid may also be used to attenuate recognition of the vector by host antibodies.

A second approach is to insert non-viral components into the capsid of the AAV. Hexa-histidine(His)-tagged design ankyrin repeat protein (DARPin), which is specific for Her2, CD4, and EpCAM has been inserted into the subunit VP2 of the AAV2 (Münch et al., 2015). Other non-viral components can be inserted into the capsid to render its stimulus responsive (Brun et al., 2017). For example, a small peptide locks consisting of tetra aspartate residue flanked by various protease cleavage sequence was inserted in the close proximity to the HSPG-binding of AAV2 (Judd et al., 2014). These peptide locks impede the vector’s ability to transduce cells until it cleaves off the capsid with extracellular proteases.

A third rational design approach uses chemical biology to make a more specific alteration to the capsid. The aldehyde tag is inserted into all three subunits of AAV2 capsid (Liu et al., 2013). Different types of molecules can be attached to the aldehyde tag. In a study, non-canonical amino acid AzK was genetically incorporated at five different surface exposed regions on the capsid. A synthetic peptide targeting αvβ3 integrin receptors was chemically conjugated into the AzK residues of the two AAV variants (T454Azk, R588AzK). The latter mutant shows efficient vector retargeting and transduction of ovarian cancer cells with high αvβ3 expression (Lee et al., 2018).

### 4.2 Roles of different promoters and virus serotypes in various types of stem cells

Transgene expression in HSC depends on the efficiency of gene transfer, the transduction process including promoter activity and transduction enhancers. This is because the expression level of transgene in HSCs is strongly controlled by the promoters. The most used promoters in vectors are cytomegalovirus (CMV), endogenous elongation factor 1 alpha (EF1a), and phosphoglycerate kinase (PGK). The potential of retroviral vector transgene delivery should be associated with the use of optimal promoters to improve the transgene expression in HSCs (Varma et al., 2011). A study by Liu clearly demonstrated that transgene in stem cells is dependent on the promoter strength (Liu et al., 2006) promoters play an important role in gene expression. Therefore, lentiviral vectors are used to examine four different promoters and their activities in cord blood HSCs.

Efficient reporter gene systems are also important for studying promoter function. GFP has emerged as an excellent reporter gene for gene transfer studies in various cell lines, including hematopoietic cells (Bierhuizen et al., 1997). The auto fluorescent nature of GFP enabled rapid identification of transduced cells by simple microscopy, greatly improving gene transfer. Furthermore, GFP does not require other gene products or co factors to produce fluorescence, all it needs for fluorescence is oxygen and blue light. The results demonstrated that promoter-dependent expression of reporter genes in the cord-blood derived HSCs (Varma et al., 2011). CMV-GFP and EF1-GFP showed the highest transduction rate in contrast, SV40-GFP and UBC-GFP showed very low transduction rate.

In another study, CMV and EF1A promoters are used to produce high levels of transfection in many cell types. Nevertheless, CMV promoter showed significantly higher transfection rates compared to EF1a promoter especially in human mesenchymal stem cells (hMSCs) (Hamann et al., 2019). This increase in transfection through CMV promoter compared to EF1a promoter may be due to increased transcription of transgene. Antonova et al. showed that CMV promoter generates more transgenic mRNAs transcripts compared to other promoters in mouse and primary human fibroblasts (Antonova et al., 2020). However, latest report shows no increase in transgenic mRNAs transcripts in human amniotic mesenchymal stem cells (hAMSCs) from the CMV compared to EF1a promoter at either 12 or 24 h after transgene delivery in any of the condition tested (Kozisek et al., 2021).

The constitutive cytomegalovirus (CMV) enhancer/chicken B-actin promoter (CAG) promoter was recently used to generate endodermal progenitor cells from hESCs by over-expression of SOX17 and SOX7 (Ca, 2008). Four overexpressed transcription factors SOX2, OCT3/4, KLF4 and c-MYC were used to reprogram somatic cells into induced pluripotent stem cells (iPSCs) using constitutively active elongation factor 1a (EF1a) promoter (Takahashi and Yamanaka, 2006). Furthermore, to monitor and track iPSCs generated from mouse fetal fibroblast, eGFP was constitutively expressed using EF1a promoter. Therefore, continuously expressed fluorescent reporter genes can be used for live imaging of hESCs *in vitro* or differentiation in animal transplantation experiments without the use of time-consuming species-species antibody labelling systems or *in situ* hybridization (Norrman et al., 2010).

Besides phosphoglycerate kinase (PGK), EF1a promoters have also been effectively used for long term transgene expression in ESCs. Both promoters have been shown to mediate stable, long-term expression of eGFPs in hESCs, whereas CMV promoter mediated only transient expression (Liu et al., 2009). Thus, EF1a and PGK promoters are more stable then CMV promoters in mouse ES cells (Wang et al., 2008). Comparative studies of CMV and EF1a promoters demonstrated that EF1a is superior to CMV promoters in undifferentiated mouse, monkey and human ESCs (Kim et al., 2007). Using EF1a promoter, a stable EF1a-eGFP hESCs were generated that able to maintain eGFP expression for up to 4 weeks in culture.

AAV vectors have been shown to be able to efficiently transduce human MSC without exposing the cells to genotoxic stresses (Pagnotto et al., 2007; Gabriel et al., 2017). A study by Chng et al. compares the transduction efficiency of AAV serotypes 1,2,3,4,5,6 and 8 in MSC and find that serotype 2 was the most effective at transducing MSCs under tested conditions (Brown et al., 2017). For instance, MSCs that overexpress BMP-7 or transforming growth factor Beta 1 following a vector transduction have been shown to stimulate osteogenesis and repair damage cartilage resulting in improved joint healing *in vivo* (Wei et al., 2017).

AAV 1,6, and 9 are the most cardiotropic, and AAV2 is the typical serotype whose biology has been most extensively studied. To assess the efficacy of the viral transduction system in pluripotent cells and cardiomyocytes derived from them, these four known serotypes were tested. This study demonstrated the superiority of AAV2 and AAV6 in an *in vitro* system (Rapti et al., 2015). It is also interesting that the efficiency of AAV2 transduction appears to saturate at higher viral genome concentration per cell, although this process can be explained by limitations in receptor binding or intracellular trafficking (Nonnenmacher and Weber, 2012).

Furthermore, a novel AAV variant, AAV1.9 has been isolated that demonstrated ∼48% improved infection efficiency at MOI 100,000. AAV1.9 contains a single R459G mutation, which lies closely to the heparin binding domain and the FGF receptor binding domain on AAV2. Although this mutant showed a slightly higher affinity for heparin compared to AAV2, it is observed similar levels of binding of AAV1.9 and AAV2 to hESCs (Asuri et al., 2012). In another study, AAV2 is highly efficient in delivering genes into human bone marrow stem cells (hBMSCs) (Yao et al., 2023). High transduction efficiencies (>80%) can be achieved by adding virus particles to cell cultures at MOI OF 10,000. When hBMSCs are infected with higher MOI (>100,000) of AAV2 or its mutant AAV2.7m8, the transduction efficiency can reach nearly 100%. However, the result suggest that genetic background of cell donor may influence AAV transduction efficiency, especially at low MOI (Yao et al., 2023).

### 4.3 Non-viral vectors

#### 4.3.1 Exosomes

Exosomes, specialized membrane-like vesicles ranging from 50 to 150 nm in size, are a new way for cells to communicate and are involved in various cellular processes such immune system, signal transduction, and antigen presentation (Liu and Su, 2019). They are part of a small fraction of extracellular vesicles (EV) and can be identified and isolated from supernatants and body fluids. Exosomes are naturally derived and possess a low toxicity profile and immunogenicity profile, have a longer half-life circulation and able to penetrate larger tissue through surface ligands and receptors (Jafari et al., 2020). They can also penetrate biological barriers such as the blood brain barrier (BBB) and the cytoplasmic membrane (Jafari et al., 2020; Liu and Su, 2019).

Since almost every eukaryotic cell can release exosomes, their content can vary a lot depending on the type of the cell they come from and their current status (Zhang et al., 2019b). Exosomes are seen as mini versions of parent cells and have a complex structure that contains various proteins, lipids, and nucleic acids. Exosomes are highly enriched with various proteins that play different roles. Tetraspanins, heat shock proteins, MVB formation proteins and annexins and Rab all play crucial roles in cell invasion, penetration, fusion, stress response, binding, antigen presentation, and membrane transport (Vlassov et al., 2012).

Multivesicular body (MVB) and late endosomes are specialized endosomal rich in intraluminal vesicles (ILVs), secreting specific proteins and lipids. ILVs are formed by inward budding of endosomal membranes and transported to the plasma membrane via cytoskeleton and microtubule network (Gurung et al., 2021). After fusion, they are exocytosed to the cell surface. Other MVBs undergo a degradation pathway where they fuse directly with lysosome or fuse with autophagosome followed by lysosomes. The endosomal sorting complex for transport (ESCRT) is a complex protein machinery that promotes MVB formation, vesicle budding, and sorting of protein cargo (Hurley, 2015; Zhang et al., 2019b). ESCRT-0 recognizes and binding ubiquitin-containing proteins to endosomal membrane domains, forming a complex with ESCRT-I and ESCRT-II. The ESCRT-III complex is involved in budding and is separated from the MVB membrane by energy supplied by Vps4 (Gurung et al., 2021; Zhang et al., 2019b).

**FIGURE 7:**
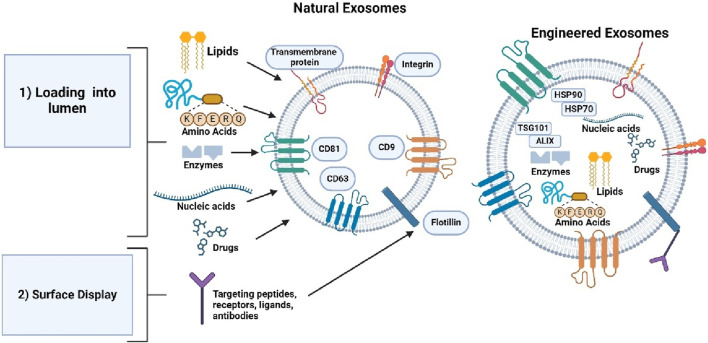
Shows the two different approaches used to generate exosomes for therapeutic applications. Created with BioRender.com.

Functional molecules can be loaded into the lumen or displayed on exosomal surface for therapeutic applications. There are two main approaches for loading and displaying molecules, parental cell-based and direct exosomal engineering. Parental cell-based involves cells as the starting material, while direct exosomal engineering uses exosomes as the initial starting material after isolation (Jafari et al., 2020). Figure 7 shows the approaches involved in generating exosomes.

Exosome engineering using the parental cell approach involves directing a protein to the exosomal surface through the exosomal signal peptide. For example, the lysosomal associated membrane protein (Lamp2b) is a protein with an exosome signal peptide. Lamp2b fusion protein is used to display the protein on the exosome surface as a target moiety, ligand, or receptor. For example, lamp2b-based fusion protein display a neuron specific targeting peptide, rabies virus glycoprotein (RVG) on the surface (Liang et al., 2021). Dendritic cells express this fusion protein, resulting in the accumulation of RVG in the neurons and brains of mice (Alvarez-Erviti et al., 2011). Lamp2b’s signal peptide can be employed to display any fusion protein on the exosome surface. Incorporating a glycosylation motif into Lamp2b-RVG may improve exosomal delivery to the neuron and glial cell compartment (Hung and Leonard, 2015; Barile and Vassalli, 2017).

Another approach in parental cell-based engineering involves loading proteins into the lumen using molecule sorting modules. Two methods are used to load proteins into exosomes based on the cell ubiquitination system of cells where uses ub-tag which removes the last two C-terminals, and short tag (WW Tag) which uses ubiquitination to load proteins into exosomes (Cheng and Schorey, 2016). The ub tag is fusion-linked to proteins such as Ag85B, ESAT 6, and tumor antigens, allowing them to be loaded into the exosome lumen in HEK293 cells. The WW tag linked to the L domain motif of Ndfip1, activates E3 ligases containing HECT domain proteins. By combining Cre recombinase with the WW tag, Ndfip1 recognizes the tag, allowing Cre’s protein to be ubiquitinated (Jafari et al., 2020). Recently, a non-functional variant HIV-1 Nef protein has been used to load specific proteins into exosomes.

### 4.4 Advantages and disadvantages of viral and non-viral vectors

Viruses can adapt to changing biological environments, making them useful in gene therapy research. Table 4 shows the advantages and disadvantages of viral vectors and nonviral vectors in therapies. The main advantage of using viral vectors in gene therapy is that they protect the transgenes against biological degradation and effectively cross cellular barriers (Ramamoorth and Narvekar, 2015). Despite initial challenges, research has continued, leading to the approval of numerous viral vector-derived therapies. These therapies are primarily directed towards the treatment of various types of cancers, monogenic diseases, infectious and cardiovascular diseases (Butt et al., 2022).

**TABLE 4:** Shows the advantages and disadvantages of viral vectors and nonviral vectors.

Vectors	Advantages	Disadvantages
Viral vectors	They have the ability to protect transgenes from biological degradation	They can trigger immune responses and inflammatory reactions
	They can cross the cellular barriers efficiently	Viral vectors are produced by complex processes
	Vectors are widely used in clinical applications	They can cause mutagenesis by inserting exogenous DNA into genome
	They can target a large number of cells	More studies are required to understand the mechanism of molecular infection of viruses
Non-viral vectors	Non-viral vectors have less immunotoxicity and are safer than viral vectors	They have a low *in vivo* gene transduction efficiency
	They can transfer larger genetic material	They contain toxicity related to the materials
	They are widely used in clinical studies	They have anatomical and cellular obstacles to the entry of nucleic acids

However, viral vectors have major drawbacks, including immunogenicity and toxicity (Mahoney et al., 2022). The first related death in the gene therapy trial was caused by an allergic reaction to the adenoviral vector. Additionally, insertional mutagenesis is another cause of concern where ectopic chromosomal insertion of viral DNA leads to tumor suppression gene disruption or activation of oncogenes resulting in malignant transformation of cells (Thomas et al., 2003). Adenovirus vectors are known to be the most immunogenic and greatest challenge that gene therapists face in using them in clinical studies (Mahoney et al., 2022).

Manufacturing procedures for viral vectors involve a wide range of approaches, mainly based on mammalian cells in adherent or suspension cell-based systems. Laboratory-scale systems containing adherent cells are difficult to expand into larger scale due to the use of a high number of flasks, roller bottles, and cell factories, which limits incubator space and increases processing time and risk due to the numerous steps involved in the aseptic processing process (van der Loo and Wright, 2016).

The nonviral vector offers a low toxicity profile and low risk of mutagenesis and is more likely to produce on a larger scale due to its simplicity of production (Ramamoorth and Narvekar, 2015). They also have non-immunogenic properties, making repeated administration feasible for therapeutics (Al-Dosari and Gao, 2009). Nonviral vectors do not contain any viral components, thus eliminating the potential risk of recombination or other safety issues. There are no restrictions on the size of the inserted DNA, and the cell entry process uses natural cellular uptake pathways compared to direct DNA injection (Collins, 1999).

Plasmid expression vectors are widely used in nonviral gene therapy studies due to their simplicity of design and amplification. They are also episomal and non-integrating, minimizing insertional mutation risk compared to viral vectors (Yin et al., 2014). The selection of an enhancer and promoter combination significantly effect on the level and duration of transgene expression (Xu et al., 2019).

Gene transfer by nonviral methods is limited by anatomical and cellular obstacles such as epithelial and endothelial cell linings and extracellular matrix (Al-Dosari and Gao, 2009). Nucleases in the blood and cellular matrix can degrade the unprotected nucleic acid administered systemically (Gascón et al., 2013). However, they may have a low transfection rate compared to viral vectors, and critical issues such as target specificity and the long time required for gene expression are being explored.

### 4.5 Application of biological vectors

After decades of research and development, viral vectors and non-vector-based gene therapies have demonstrated promising clinical results with numerous products approved for the treatment of a variety of conditions, including cancer, infectious diseases, and monogenic diseases (Zhao et al., 2021b). Furthermore, several ongoing clinical trials are being conducted to further enhance their therapeutic capabilities. Table 5 summarizes the list of examples of viral vectors applications in the direction of stem cell differentiation.

**TABLE 5:** Summarizes the list examples of viral vectors application in the direction of stem cell differentiation.

Viral vectors	Study disease	Key findings	Animal model	References
Herpes simplex virus vector (HSV)	Colon cancer treatment	CRISPR/Cas9 was used to create an HSV-based virus, where coding region ICP34.5 was replaced by therapeutic genes murine (IL12, p40-p35) and C-X-C motif chemokine ligand 11, and the deletion of ICP47 gene. The combination of IL12 and CXCL11 with oncolytic virus have a promising treatment in colon cancer	N/A	Zhang et al. (2023)
	Autosomal recessive congenital ichthyosis (ARCI)	The main cause of ARCI is by germline mutation in a gene that codes for a specific enzyme, TGM1. This study focused on the feasibility of delivering human TGM1 using KB105, a full-length gene therapy vector encoding human TGM1	BALB/c mice	Freedman et al. (2021)
	Esophageal carcinoma (EC)	The triple mutation, conditionally reproducing HSV-1 has a high safety feature that enables it to significantly inhibit the growth of tumour cells. G47Δ is safe when administrated at high dose through intramuscularly and orally. Therefore, intramuscular injection is a viable and practical therapeutic option for treating esophageal cancer	Six-week-old athymic mice (BALB/c and nu/nu)	Yajima et al. (2021)
Lentiviral vectors	Pompe disease	Lentiviral vectors were fused with insulin like growth factor 2 (IGF2) to a codon optimized version of GAA (LV-IGF2.GAAco) which improve the cellular uptake by cation-independent mannose 6-phosphate/IGF2 (CI-M6P/IGF2) receptor LV-IGF2.GAAco effective in treating central nervous system (CNS) by normalising glycogen level and achieved vector copy number between 0.5 and 3 for neuroinflammation	N/A	Liang et al. (2022)
	Hemophilia A	Intraosseous (IO) delivery of factor VIII (FVIII, gene 8) lentiviral vector via megakaryocytes -specific promoter (Gp1bα) partially corrected the bleeding phenotype in Hemophilia A (HemA) mice	C57BL/6 mice	Joo et al. (2022)
	Duchenne Muscular Dystrophy (DMD)	Vector was designed to contain a muscle-specific promoter and sequence optimized full-length dystrophin to restrict the expression of dystrophins for differentiation of myotubes and myofibers	Muscular dystrophy X-linked mouse (mdx)	Meng et al. (2022)
	Bladder cancer	Examined the efficacy of lentiviral vector expressing murine IFNα and the expression is exhibit by intravesical instillation through transducing murine BLCA cell lines and bladder urothelium	N/A	Mokkapati et al. (2022)
Adenoviral vector	Leydig cell hypoplasia	AAV8 identified as an effective vector to stimulate exogenous luteinizing hormone/choriogonadotrophin receptor (Lhcgr) expression in Leydig progenitor cells via interstitial injection. This results in the partial restoration of sexual development and restore spematogenicity and successful production of fertile progeny	Male C57BL/6 mice	Xia et al. (2022)
	Age-related macular degeneration (AMD)	Vector from a chimp (AdC68-PFC) that included 3 genes such as pigment endothelial-derived factor (PGDF), soluble fms-like tyrosine kinase-1 and soluble form of CD59. AdC68-PFC demonstrated preventive and therapeutic efficacy after intravitreal injection. The safety test revealed no *in vivo* toxicities of adenovirus in the murine eyes	Male C57BL/6J mice	Wei-Zhang et al. (2023)
	Influenza A infection	Non-replicating adenovirus vector that encodes a secreted form of H1HA was developed to assess HA stalk-directed immunity. Ad5_H1 vaccine was evaluated in mice to determine whether it provides broad cross-reactivity against homologous and heterologous lethal threats in a single-dose	Female BALB/cJ mice	Bliss et al. (2022)
	Recessive dystrophic epidermolysis bullosa (RDEB)	Ad vectors are developed for the delivery of CRISPR-Cas9 components to correct a mutation that induces RDEB and assessed their ability to treat this condition *in vivo* by direct application to a humanized RDEB skin model	C57BL/6 mice	García et al. (2022)

Lentiviral vectors are being used to study and treat Duchenne Muscular dystrophy (DMD), a genetic disorder that is X-linked and caused by mutations in the gene (Duan et al., 2021). The primary treatment strategy is the restoration of dystrophin protein in affected muscles. However, systemic delivery to skeletal muscle is still difficult due to the high number of cells needed and inability to target skeletal muscle after intra-arterial or intravenous administration. Satellite cell-derived myoblasts can be used to treat key muscles in patients with DMD, such as the thenar muscles responsible for fine movements. Gene editing can correct DMD precisely, but this approach is highly mutation-dependent and unlikely to be effective for complete cDNA repair (Young et al., 2016; Amoasii et al., 2017). However, viral vectors introduced dystrophin coding sequence into cells and may be applicable to most patients with a wide range of mutations (Meng et al., 2022).

Lentiviral vectors (LV) enter the host genome and provide a sustained expression of the transgene. LVs are capable of transducing muscle satellite cells *in vivo*, which could be a long-term treatment for DMD characterized by myofiber necrosis (Kimura et al., 2010). LVs have been demonstrated to be safe and effective in clinical studies (Milone and O’Doherty, 2018), both *ex vivo* and *in vivo* plus able to package full length dystrophin (Counsell et al., 2017). When the original SSFV promoter was replaced with CK9 promoter to drive the natural form of dystrophin (nFLDys) CDNA in lentiviral vectors, the restored dystrophin was found to be lower than normal level in both *in vitro* and *in vivo*. Replacing the insert with sequence-optimized full-length dystrophins (soFLDys) resulted in a 6-fold increase in protein expression of dystrophin *in vitro* and 2.8-fold increase in sarcolemma dystrophin intensities in donor fibers *in vivo* compared to nFLDys. SoFLDys-driven protein expression improved not only at translational levels but also at transcriptional levels (Meng et al., 2022).

Besides viral vectors, non-viral vectors have been utilized for clinical studies as they are less immunogenic and toxic towards the cells. Table 6 summarizes the list of examples of the non-viral vectors application in the direction of stem cell differentiation. Chimeric antigen receptor T(CAR-T) cells are effective in treating blood cancer like chemo-resistant or relapse leukemia (Porter et al., 2011). However, they struggle to target solid tumours due to low levels of tumour-associated antigen (TAA) and lack of tumour penetration (Zhang et al., 2018). A study has modified the CAR molecule to recognize TAAs using artificial single chain fragment variable (scFv) or a specific binding domain. PiggyBac mediated CAR-T cells demonstrated anti-tumor activity *in vitro* and clinically in EPHB4-positive solid tumours.

**TABLE 6:** Summarizes the list of examples of non-viral vectors applications in the direction of stem cell differentiation.

Non-viral vectors	Study disease	Key findings	Animal models	References
Piggy bac transposons	Acute lymphoblastic leukemia (ALL)	This study examined the efficacy and adverse effects of CD19 chimeric antigen receptor(CAR) T cells against ALL in the CNS using a xenograft mouse model. Mouse model has been administrated either by intravenous or intracerebroventricular delivery of CAR T cells. Partial effects were observed when piggy bac CD19 CAR T cells were injected through intravenously	8–10-weeks old male NOG mice	Tanaka et al. (2020)
	Hepatocellular carcinoma (HCC)	A novel electroporation approach was designed for PB transposon to generate GPC3-specific CAR-T cells for HCC therapy. These cells able to reduce tumour while simultaneously secreting high level of interferon-γ. Findings suggest that PB system based GPC3-CAR-T cells may be a promising strategy for HCC patients	Huh-7 Xenograft mouse model	Wang et al. (2020)
	Rhabdomyosarcoma	Ephrin type-B receptor 4 (EPHB4) has been responsible and expressed in tumours which makes a good target for CAR-T cells. PB transposon was used to generate EPHB4-CAR-T cells which allowed for sufficient T cell proliferation. These cells able to stop positive tumour cells expressing EPHB4 without activating cell proliferation	SCID beige mice	Kubo et al. (2021)
mRNA	Hereditary tyrosinemia type 1	Hereditary tyrosinemia type 1 is caused by lack of fumarylacetoacetate hydrolase (FAH). Repeated administration of lipid nanoparticle -based FAH mRNAs into mouse model resulted in synthesis if FAH protein, maintain body weight and a normalized pathologic elevation of post nitisinone cessation and also decrease the risk of early death	Fah-deficient mouse model (Fah1R Tyrc/RJ)	Cacicedo et al. (2022)
	Type 2 diabetes (T2D) and non-alcoholic steatohepatis (NASH)	Fibroblast growth factor 21 (FGF21) has been identified as a potential therapeutic target in the treatment of T2D and NASH. The effectiveness of mRNAs was evaluated by 2 weeks of repeated subcutaneous administration in mice. This shows significant reductions in body weight and plasma insulin levels as well as hepatic steatosis	C57Bl/6 mice	Bartesaghi et al. (2022)
	Breast cancer	Trastuzumab is the first approved monoclonal antibody against human epidermal growth receptor-2 (HER-2) breast cancer. This antibody significantly limits the therapeutic results. Thus, tumour microenvironment (TME) pH-responsive nanoparticles have been developed to deliver systemic mRNAs to reverse the trastuzumab resistance of breast cancer	Female BALB/c mice	Dong et al. (2023)
Exosomes	Myocardial infarction (MI)	In this study, microfluidics is used to create artificial stem cells that could release Tβ4-exosomes. By encapsulating these specific exosomes in microspheres, this could replicate the paracrine as well as biological activity of stem cells. The results demonstrated that Tβ4-ASCs are highly effective in promoting collateralization at peripheral area of myocardial infarcted site and their therapeutic effect is better than direct exosome injection. It can also promote the formation of post-myocardial collateral circulation and alternative way to clinical revascularization	C57BL/6 mice	Chen et al. (2022)
	Ischemic stroke	The objective of this study was to determine whether adhesive hyaluronic acid(HAD) hydrogel loading exosomes derived from NSCs could be sued to facilitate the recovery of patients with ischemic stroke. These exosomes help to stimulate neurogenesis and migratory pattern in NSCs	C57BL/6J adult male and female mice	Gu et al. (2023)
	Intracerebral hemorrhage (ICH)	This study demonstrated that the use of exosomes obtained from young healthy human plasma with typical exosomes characteristics could facilitate functional recovery in ICH mice. Furthermore, the administration of exosomes significantly improved the behavioural recovery by reducing brain damage and cell ferroptosis	C57BL/6 male mice	Yang et al. (2023)

Ephrin type-B receptor 4 (EPHB4), which belongs to receptor tyrosine kinases (RTKs) expressed extensively in variety of tumours such as lung cancer, colorectal cancer, and malignant soft tissue sarcoma, including rhabdomyosarcoma (RMS) (Kumar et al., 2009; Randolph et al., 2017). The binding to interacting ligand results in a two-way signaling cascade between receptor-expressing and ligand presenting cells (Chen et al., 2019). Activation of ligand-independent EPHB4 can lead to cell proliferation and tumor transformation, while ligand-dependent activation induces cell death in certain tumours (Aslam et al., 2014).

The study found that the expression of mRNA and proteins in RMS tumor is higher than in normal skeletal muscles (Aslam et al., 2014). Despite the interaction of human EPHRIN B2 with murine protein, no adverse effects were observed in mouse model, dispelling the safety concerns (Kubo et al., 2021). P3F maybe linked to an immune escape mechanism in addition to its proliferation, motility and suppression of differentiation in RMS (Kikuchi et al., 2008). The study found that P3F knockdown did not suppress PD-L1 expression in Rh30 cells after 24 h of co-culture with EPHB4 CAR-T cells. Further modification of EPHB4-CAR-T cells may be necessary to improve its efficacy and safety in clinical therapeutics (Kubo et al., 2021).

## 5 Safety and efficacy issues of design molecules

Despite all the advancements in stem cell differentiation, there are concerns about the safety and efficacy of design molecules. For aptamer, the constructs has slightly inferior structural and functional properties compared to native meniscus (Li et al., 2022). Apt19S alone is not sufficient for osteogenic differentiation, as osteoinductive growth factor BMP2 is needed (Sun et al., 2021). Besides, rapid clearance of aptamer may lead to loss of targeting efficacy in the human body (Byun, 2021), and RBM-007 aptamers were also highlighted to have uncertain efficacy which needs further evaluation in human studies (Kimura et al., 2021). Also, the use of unnatural nucleotides or bulky moieties for chemical modifications can evoke safety risks, so should be avoided when necessary (Ni et al., 2021).

For phage, short clearance rate poses challenges in research studies since the quick elimination of phage from the system reduces the phage efficacy (Caflisch et al., 2019). Although the use of M13 phage displaying functional peptides seems promising in improving engraftment and angiogenesis (Jang et al., 2020), had also noted some unexpected *in vitro* results of the pre-treated hCPCs. Plus, there are concerns of side effects and development of phage-resistant bacteria due to genetic mutation (Caflisch et al., 2019). Most phage genes are also still of unknown function which may cause undesirable events (Monteiro et al., 2019), and the methods of administration and dose of bacteriophage used are in need to be deliberately considered (Podlacha et al., 2021). Additionally (Peng and Chen, 2021), had highlighted the possibility of inducing chemical changes towards chemically modified phage which could interfere in the phage functionality. Hence, these issues highlight the importance of more evaluations and studies to be carried out to secure its safety and efficacy.

Viral vectors which have their origin in wild type viruses, can be used as treatments or prophylactics (Shirley et al., 2020). Effective prophylaxis may be crucial in controlling environmental risk assessment. It is important to assess if the alteration will lead to a lower sensitivity of viral vector to prophylaxis that is effective against the wild type (Reuter et al., 2012; Baldo et al., 2013). Characterizing the genetically modified virus (GMV) is crucial in identifying hazards. If the virus is attenuated through modification, the risk group of the viral vector maybe lower than the wild-type virus from which it is derived (Baldo et al., 2013). Transgene characteristics must also be considered when assigning recombinant vectors to risk groups. One method of effectively attenuating the viral vector is minimizing self-propagation in target cells while maintaining the ability to introduce the gene of interest (Rakowska et al., 2021).

Non-viral vector are used to prevent endonuclease destruction of the genetic material, which allowing nuclear absorption and vector unpacking (Zu and Gao, 2021). However, their larger particles can hinder their effectiveness, as DNA must be transported into the nucleus for therapeutic impact (Torres-Vanegas et al., 2021). Understanding the physiochemical and biological characteristics of genetic material and the carrier, and the molecular mechanistic understanding of vector-induced transfection, is crucial for creating and effective and safe non-viral vectors (Jones et al., 2013). Toxicology should be considered, as nanoparticles may cause an immunological response, despite being less immunotoxic than viral vectors. Additionally, the pH and ionic strength of the formulation buffer can affect vector transfection efficiencies. Gene therapy formulations are often stored by freezing or refrigeration which can increase temperature before delivery and reduce efficacy (Kafetzis et al., 2023).

## 6 Conclusion

Regenerative medicine is a field that uses stem cell technologies, and physical and chemical interventions to treat diseases. However, challenges remain, such as obtaining stem cells and controlling cell biological behavior. Techniques and technologies have been developed to direct stem cell differentiation, including designing molecules like aptamers, phages, and biological vectors.

Aptamers demonstrate robust plasticity and broad transformation potential for applied regenerative medicine research (Luo et al., 2022). However, challenges remain, such as cost control, developing new types of aptamers, increasing screening efficiencies, and optimizing connections with other biomedical materials. Future research should focus on solving the above issues to advance the clinical application of targeted aptamer-based therapeutic strategies. Aptamer-based targeted therapy is an approach that may overcome the limitations of current therapeutic strategies, so this area deserves further research attention (Liu et al., 2023).

In the era of synthetic biology, engineered phages have improved therapeutic properties, with the ability to carry long chain DNA or display protein, making them ideal for vaccine development and gene therapy (Tao et al., 2019). The TXTL cell-free synthesis method has made significant strides in recent years, and the CRISPR-Cas system has demonstrated its usefulness for various application due to its non-propagative and genome independent properties (Azam et al., 2021).

Advances in vector design have enabled vectors with better systemic distribution and spatial control for gene expression, driving gene therapy into clinical trials for a wide range of diseases (Nelson et al., 2020). A viral vector should meet safety, easy manufacturing, efficient reach to target cells, and reproducibility in animal model before being used in human clinical trials (Nance and Duan, 2018). Appropriate laboratory and manufacturing practices are necessary for bringing viral therapeutics into clinics with quality assurance required at every stage of production, validation, and storage.
